# Validity of stem cell-loaded scaffolds to facilitate endometrial regeneration and restore fertility: a systematic review and meta-analysis

**DOI:** 10.3389/fendo.2024.1397783

**Published:** 2024-05-23

**Authors:** Qiao-yi Huang, Hui-da Zheng, Qi-yang Shi, Jian-hua Xu

**Affiliations:** ^1^ Department of Gynaecology and Obstetrics, Second Affiliated Hospital of Fujian Medical University, Quanzhou, China; ^2^ Department of Gastrointestinal Surgery, Second Affiliated Hospital of Fujian Medical University, Quanzhou, China

**Keywords:** meta-analysis, biocompatible materials, scaffolds, stem cells, intrauterine adhesion

## Abstract

**Objective:**

Various stem cell-loaded scaffolds have demonstrated promising endometrial regeneration and fertility restoration. This study aimed to evaluate the efficacy of stem cell-loaded scaffolds in treating uterine injury in animal models.

**Methods:**

The PubMed, Embase, Scopus, and Web of Science databases were systematically searched. Data were extracted and analyzed using Review Manager version 5.4. Improvements in endometrial thickness, endometrial glands, fibrotic area, and number of gestational sacs/implanted embryos were compared after transplantation in the stem cell-loaded scaffolds and scaffold-only group. The standardized mean difference (SMD) and confidence interval (CI) were calculated using forest plots.

**Results:**

Thirteen studies qualified for meta-analysis. Overall, compared to the scaffold groups, stem cell-loaded scaffolds significantly increased endometrial thickness (SMD = 1.99, 95% CI: 1.54 to 2.44, P < 0.00001; I² = 16%) and the number of endometrial glands (SMD = 1.93, 95% CI: 1.45 to 2.41, P < 0.00001; I² = 0). Moreover, stem cell-loaded scaffolds present a prominent effect on improving fibrosis area (SMD = −2.50, 95% CI: –3.07 to –1.93, P < 0.00001; I² = 36%) and fertility (SMD = 3.34, 95% CI: 1.58 to 5.09, P = 0.0002; I² = 83%). Significant heterogeneity among studies was observed, and further subgroup and sensitivity analyses identified the source of heterogeneity. Moreover, stem cell-loaded scaffolds exhibited lower inflammation levels and higher angiogenesis, and cell proliferation after transplantation.

**Conclusion:**

The evidence indicates that stem cell-loaded scaffolds were more effective in promoting endometrial repair and restoring fertility than the scaffold-only groups. The limitations of the small sample sizes should be considered when interpreting the results. Thus, larger animal studies and clinical trials are needed for further investigation.

**Systematic review registration:**

https://www.crd.york.ac.uk/PROSPERO, identifier CRD42024493132.

## 1 Introduction

Intrauterine adhesion (IUA), characterized by endometrial fibrosis, is the primary cause of refractory uterine infertility. The risk factors of IUA are repeated intrauterine manipulations, inflammation, ischemia, and infections. These factors may trigger an aberrant cellular response and insensitivity to estrogen and progesterone, disrupting endometrial homeostasis (1). Patients with IUA mainly present with menstrual abnormalities, recurrent spontaneous abortions, and placental abnormalities (2). Surgical removal of adhesive tissue is a clinically recommended method for IUA. Although this procedure greatly improves menstruation and increases pregnancy rates, the disease recurrence rate can reach 62% (3). Another routine strategy is introducing a physical barrier into the uterine cavity after adhesiolysis. However, solid barriers, such as intrauterine devices and catheters, frequently cause irregular vaginal bleeding and local inflammation. Hydrogels and other semi-solid barriers have limited healing benefits. Furthermore, drugs that improve blood flow, such as metformin, have lower efficacy. Therefore, an unmet need exists to develop effective treatments for IUA.

Mesenchymal stem cells (MSCs) are multi-potent cells with a self-renewal and multi-lineage differentiation potentials. They exist widely in various tissues, such as adipose tissue, menstrual fluid, placenta, umbilical cord, amniotic fluid, and bone marrow (4). MSCs have many advantages, including low immunogenicity, homing to the site of injury, anti-inflammatory activity, and paracrine profiles (5, 6). Numerous animal experiments have demonstrated that MSCs have a great therapeutic effect in attenuating endometrial fibrosis, increasing the number of endometrial glands, and promoting vascular regeneration (7–9). Furthermore, MSC transplantation for IUA has entered phase I clinical trials. The results indicate that MSCs can repair a damaged uterus and increase ongoing pregnancy rates without serious adverse events (10, 11). However, additional research has observed that only a few MSCs engrafted into the endometrium two or three weeks after intrauterine injections (12). Multiple factors co-regulate the ability of stem cells to participate in endometrial repair, including post-implantation cell survival and the presence of proper stimuli in the surrounding microenvironment. Engineering-controlled and predictable cell transport strategies to guide stem cell responses are crucial.

Many researchers have focused on biomaterials in search of methods to achieve long-term retention of stem cells *in vivo* and the sustained release of their derivatives. Biomaterials have been used to load MSCs, drugs, and growth factors or to construct *in situ* delivery systems due to their biocompatibility, hydrophilicity, stability, and degradability. Scaffolds provide a support network for carriers, preserving their structural integrity and activity. Moreover, cells and drugs are released as the scaffold degrades, triggering tissue reactions including vascularization, differentiation, and cellular infiltration (13). Another critical aspect is that scaffolds can be engineered into porous scaffolds and grafts that mimic natural endometrial tissue and microenvironment. This microenvironment can simulate natural cell-cell, cellular extracellular matrix, and cell-soluble factor interactions (14). Current studies have demonstrated that various bio-scaffolds, such as hydrogels, collagen, and poly (glycerol sebacate), combined with MSCs can effectively repair the morphology and function of the endometrium (9, 15, 16). In addition, 3D-printed biomaterials have been widely used to mimic histological architecture and functions of endometrial tissue. On the one hand, 3D-printed scaffolds could construct a cell-laden scaffolds with ideal spatial distribution to form the ultrastructure of tissues (17). On the other hand, cells recruited into 3D scaffolds can form their own extracellular matrix (ECM) and achieve further refined tissue remodeling by secreting matrix proteins (18). After endometrial injury, lack of local ECM structure makes it difficult for tissue remodeling to repair the original tissue structure, and the structure and function of the endometrium may not be effectively restored. In this case, 3D-printed biomaterials can produce a more precise and biomimetic physiological environment, resulting in better curative effect.

In the current study, few systematic reviews and meta-analyses evaluate the efficacy and safety of stem cell-loaded scaffolds for treating IUA. Our primary aim was to investigate the reported trials of stem cells pre-seeded with different scaffolds used in animal models of IUA using a meta-analysis approach, focusing on their efficacy in treating endometrial regeneration, including changes in endometrial thickness, number of endometrial glands, fibrosis area, and number of gestational sacs/implanted embryos. Furthermore, we aimed to explore potential stem cell-loaded scaffolds therapy mechanisms, such as anti-inflammatory effects, angiogenesis, and cell proliferation.

## 2 Methods

This systematic review and meta-analysis were conducted per the Preferred Reporting Items for Systematic Reviews and Meta-Analyses 2020 statement (PRISMA 2020, **Additional File 1**) (19), and registered on the International Prospective Register of Systematic Reviews (PROSPERO, CRD42024493132) website. No ethical approval was required because the meta-analysis was based on published articles.

### 2.1 Search strategy

A systematic literature search was using the PubMed, Embase, Web of Science, and Scopus databases from inception to October 17, 2023. Articles containing the following Medical Subject Headings (MeSH) or free text, separately and in combination, were included: gynatresia (Mesh), biocompatible materials (Mesh), stem cells (Mesh), Asherman syndrome (free text), intrauterine adhesion (free text), and scaffolds (free text). A detailed search strategy is provided in **Additional File 2**. According to this search strategy, all retrieved articles were evaluated against the exclusion criteria.

### 2.2 Eligibility criteria

All the studies that directly compare the efficacy of stem cell-loaded scaffolds group versus scaffold group in treating endometrial injury were accessed. Two reviewers (QY H and HD Z) independently screened the titles and abstracts of articles identified by the electronic searches and excluded duplicate and irrelevant studies. Reviews, meta-analysis, editorials, letters, and meeting abstract were not eligible. Once two reviewers identified potentially included studies, they obtained and screened full text articles. Only studies on animal models were included. Subsequently, two reviewers examined the intervention descriptions to determine intervention types were eligible for analysis. Studies without a control group were not eligible, nor were those performing combined therapies.

Lack of data under the outcomes of interest warranted the exclusion from the present analysis (**Table 1**). When agreement was elusive, two reviewers resolved discrepancies through consensus. If this was not possible, a third reviewer (JH X) consulted for final judgment regarding any disagreements.

**Table 1:** Inclusion and exclusion criteria.

Eligibility criteria
Inclusion criteria
(1) original studies on animal models of endometrial injury
(2) studies that used stem cell-loaded scaffolds as the experimental group and scaffold-only (cell-free) as the negative control
(3) studies with at least one outcome of endometrial thickness, the number of endometrial glands, fibrosis area, and number of gestational sacs/implanted embryos
Exclusion criteria
(1) duplicate studies in the five databases
(2) studies on non-original articles, such as reviews, meta-analyses, case reports, letters to the editor, editorial commentary, conference abstracts, surveys, or satisfaction studies
(3) despite being consistent with MeSH terms and free text, the content was not specific to stem cell-loaded scaffolds therapy for endometrial injury
(4) studies without a control group
(5) studies that were conducted *in vitro*, *in silico* only, and human trials
(6) studies that could not find the full text or extract data

### 2.3 Data abstraction

Qualitative data were extracted by two independent reviewers (QY H and HD Z) from the full texts of the included studies. Similar to study selection, any discrepancies were discussed and submitted to a third reviewer (JH X) for confirmation. The following information was extracted from each qualified study: first author, publication year, animal species, number of samples, modeling methods of endometrial injury, stem cell origin, stem cell number/volume, intervention and control groups, scaffold types, treatment duration, endometrial thickness, number of endometrial glands, endometrial fibrosis area, number of gestational sacs/implanted embryos, interleukin-1β (IL-1β), interleukin-6 (IL-6), vascular endothelial growth factor (VEGF), CD31, Ki-67, and insulin-like growth factor-1 (IGF-1) levels.

The following characteristics were analyzed. For endometrial regeneration, the endometrial thickness, number of endometrial glands, and the area of uterine fibrosis were analyzed. Endometrial thickness and number of glands were examined by hematoxylin and eosin staining, and endometrial fibrosis was assessed by Masson staining. After staining, endometrial thickness and morphology were examined under a light microscope. Gland numbers were counted to determine their abundance in uterine. Collagen fibers appeared blue under the microscope. The percentage of the fibrotic area was calculated as the area of fibrosis or collagen fibers relative to the total endometrial area of view. For fertility, the number of gestational sacs or implanted embryos in each uterine horn were counted and analyzed. Regarding the inflammatory expression, IL-1β and IL-6 levels were analyzed. The VEGF and CD31 levels reflect the extent of angiogenesis. Cell proliferation was represented by the Ki-67 and IGF-1 levels. If an article used more than one experiment to quantify cytokine expression levels, polymerase chain reaction and immunohistochemistry staining were prioritized, followed by enzyme-linked immunosorbent assay and immunofluorescence staining. All original data described in the included studies were prioritized for extraction. If only figures were presented, two reviewers independently used PlotDigitizer Online APP (https://plotdigitizer.com/app) to extract data and compute the means.

### 2.4 Quality assessment

The quality of each included study was evaluated by two independent authors according to the Collaborative Approach to Meta-Analysis and Review of Animal Data from Experimental Studies (CAMARADES) checklist with minor modifications (20). Each “yes” of the following criteria was given for score 1, while “no” or “unclear response” carried no weight (score 0). The 10-item checklist included: (1) peer-reviewed publication, (2) statement of control of temperature, (3) random allocation to treatment or control, (4) blind established model, (5) blinded assessment of outcome, (6) use of anesthetic in an animal model where necessary throughout the study, (7) appropriate animal model, (8) sample size calculation, (9) compliance with animal welfare regulations, and (10) statement of potential conflict of interests. Based on a total score of 10, studies with a score of 0–3 were recognized as high risk, 4–6 as medium risk, and 7–10 as low risk. Similarly, any discrepancies were discussed, and a consensus between the authors.

### 2.5 Bias risk assessment

The Systematic Review Centre for Laboratory Animal Experimentation (SYRCLE) tool was used to assess the risk of bias in each study (21). The following bias were considered in this evaluation tools: selection bias (random sequence generation, baseline characteristics, allocation concealment), detection bias (random outcome assessment, blinding), performance (random housing, blinding), attrition bias (incomplete outcome data), reporting bias (selective outcome reporting), and other bias from other sources. Two reviewers independently conducted the work. Again, two reviewers resolved divergence of opinion through consensus.

### 2.6 Statistical analysis

Review Manager 5.4 was used to analyze and manage data. All results (endometrial thickness; number of endometrial glands; endometrial fibrosis area; number of gestational sacs/implanted embryos; and IL-1β, IL-6, VEGF, CD31, Ki-67, and IGF-1 levels) were continuous variables. The standardized mean difference (SMD) and related 95% confidence interval (CI) were calculated to compare the effect between the stem cell-loaded scaffolds and scaffold-only groups. Forest plots were constructed to show the SMD and 95% CI for each study and the pooled mean difference by combining the eligible studies. All statistical tests were two-sided, and statistical significance was considered at a P-value <  0.05. The I^2^ statistic was applied to assess heterogeneity among studies. I^2^ values of < 50%, 50–70%, and > 70% were defined as low, moderate, and high inconsistencies, respectively. When I^2^ < 50%, a fixed-effects model was used. If heterogeneity was present (I^2^ > 50%), a random-effects model was adopted. Subgroup analysis and sensitivity analyses were performed to identify the sources of heterogeneity. Additionally, a funnel plot was generated to visually examine publication bias.

## 3 Results

### 3.1 Study selection

**Figure 1:**
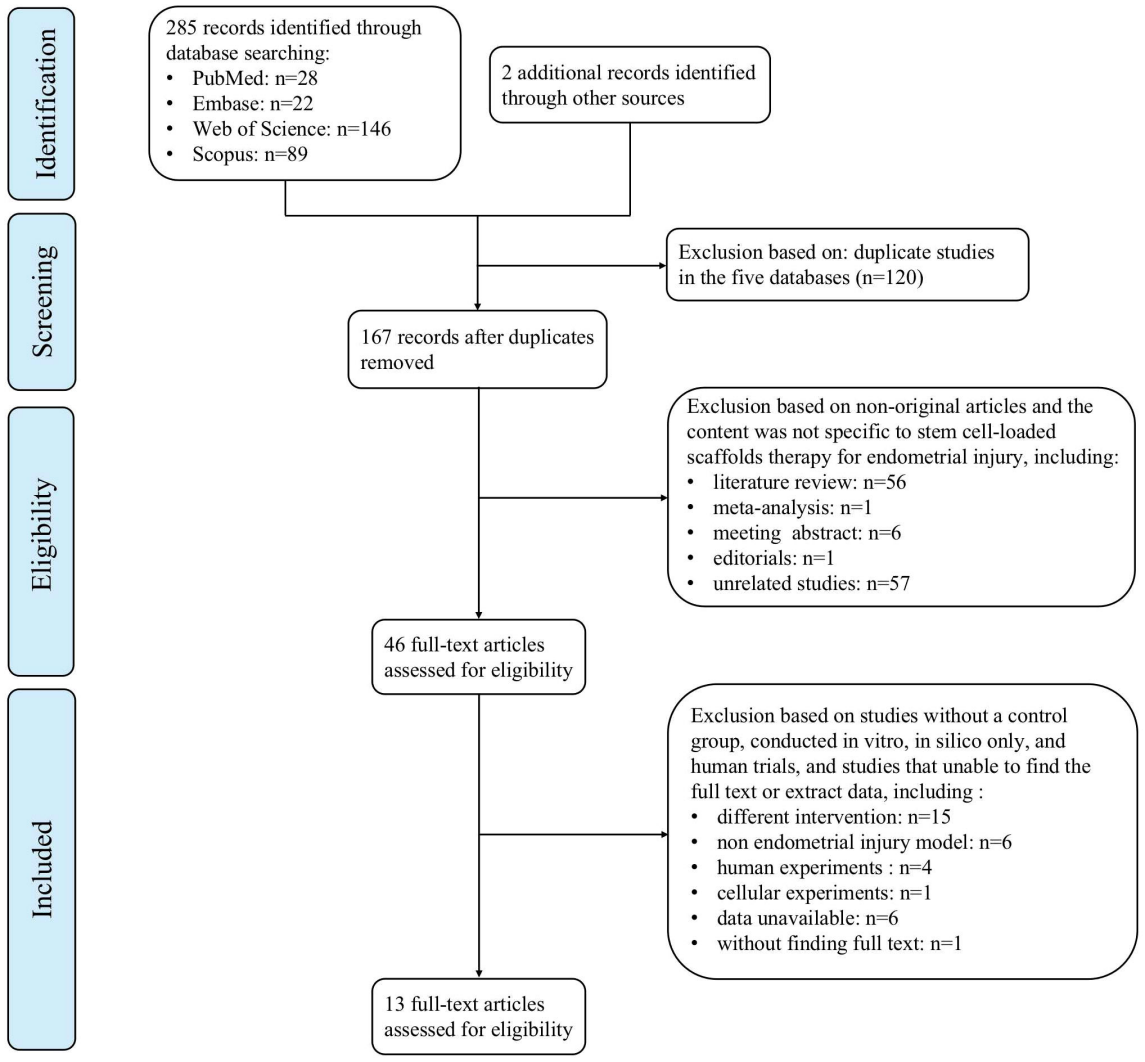
PRISMA flowchart for systematic review and study selection.

We identified 287 articles by the literature search, including 28 from PubMed, 22 from Embase, 146 from Web of Science, 89 from the Scopus databases, and two from other sources. All retrieved articles were imported into EndNote X9 (Clarivate Analytics, Philadelphia, PA, USA) to identify 120 duplicates found. After screening of titles and abstracts, 64 non-original articles and 57 unrelated studies were removed. Following full-text assessment, 32 studies were excluded mostly because “intervention did not include both stem cell-loaded scaffolds group and scaffold-only group” (n = 15), “non endometrial injury model” (n = 6), “data unavailable” (n = 6), and “human or cellular experiments” (n = 5). Finally, 13 studies were included in the meta-analysis. **Figure 1** illustrates the PRISMA flowchart.

### 3.2 Included study characteristics

**Table 2** presents the primary characteristics of the studies included in the meta-analysis. Thirteen included studies used three different animal species. The most commonly used species were SpragueDawley (SD) rats (10 articles), followed by mice (two articles), and rhesus monkeys (one article). One study focused on ICR mice, while the other focused on C57BL/6 mice. All studies explained the methods for establishing models of endometrial injury in detail: seven studies used mechanical injury, two studies used endometrium removal, two used ethanol injection, one used the mechanical injury and 95% ethanol injection, and one used mechanical injury and lipopolysaccharide infection. After successful modeling, MSCs and scaffolds were performed for treatment. Most MSCs were derived from heterologous MSCs (nine articles), followed by allogeneic MSCs (three articles) and autologous MSCs (one articles). Different types of MSCs were adopted, including human umbilical cord MSCs (three articles), rat bone marrow MSCs (three articles), human menstrual blood MSCs (two articles), and human-induced MSCs, human amniotic MSCs, human placenta-derived MSCs, human endometrium-derived MSCs, and autologous adipose-derived MSCs, one article each. Regarding the scaffold materials, one study used an elastic poly (glycerol sebacate) (PGS) scaffold, and two used a collagen scaffold, and gel scaffolds were used in the remaining ten studies. MSCs and scaffolds have been transplanted via intrauterine injection in most studies. In addition, the outcome assessment time points for treatment varied significantly, varying from one to three months. Endometrial thickness was reported in 12 studies, number of endometrial glands were reported in 11 studies, the area of endometrial fibrosis was reported in nine studies, and the number of gestational sacs/implanted embryos was reported in seven studies.

**Table 2:** Characteristics of the included studies.

Study	Year	Animal species	Exp (n) / Con (n)	Modeling	Stem cells	Stem cells number/ volume	Type of scaffolds	Duration of treatment
Yang et al (22)	2017	SD rat (8-week-old)	6/6; 8/8	mechanical damage	BMSCs	8 × 10 ^5^ / 200ul	hydrogel pluronic F-127	8 weeks
Xiao et al (15)	2019	SD rat	3/3	mechanical damage	BMSCs	6-7 × 10^5^ cells cm^−2^ scaffold / 50ul	PGS scaffold	30 days; 90 days
Chen et al (23)	2020	SD rat (6- to 8week-old)	5/5	removal of endometrium	MBMSCs	5 × 10^7^	collagen scaffolds	7 days; 28 days
Ji et al (18)	2020	SD rat	6/6; 15/15	scrape of endometrium	hiMSCs	1 × 10^6^ cells/ml	3D-printed hydrogel scaffold	1 month
Xu et al (24)	2021	SD rat	6/6; 12/12; 3/3	injection of ethanol	hUCMSCs	2 × 10^7^ cells ml-1 / 25ul	Matrigel microspheres	21 days
Hu et al (25)	2022	SD rat (8- to 12- week- old)	3/6	mechanical injury and LPS infectious	MBMSCs	2 × 10^7^ cells ml-1 / 50ul	collagen scaffold	90 days
Huang et al (26)	2022	SD rat	3/3; 4/4	injection of 95% ethanol	hAMSCs	1 × 10^7^ / 100ul	PPCNg	14 days
Lin et al (27)	2022	ICR mice (8-week-old)	6/6; 8/8	mechanical injury and injection of 95% ethanol	hPMSCs	2 × 10^5^ / 25ul	HA-GEL	7 days
Lv et al (28)	2022	SD rat (8-week-old)	6/5	mechanical damage	BMSCs	1×10^9^ L^-1^ / 200ul	Pluronic F-127 hydrogel	7 days
Zhang et al (9)	2023	SD rat (8-week-old)	15/15; 10/10	mechanical scratching	hUCMSCs	5 × 10^6^ cells	HACHO / Gel-ADH hydrogel	3 estrous cycles
Liu et al (29)	2023	SD rat (8-week-old)	5/5	mechanical damage	hEMSCs	2 × 10^6^ / 40μL	HA-GEL	60 days; 90 days
Zhao et al (30)	2021	C57BL/6 mice (6- to 7week-old)	10 vs 10	mechanical damage	ADSCs	5 × 10^6^ / 10μl	ShakeGe l™3D	7 days
Wang et al (31)	2020	rhesus monkey (6- to 7year-old)	3 vs 3	mechanical injury	hUCMSCs	1-2×10^7^ cells / 50μl	autocross linked HA-Gel	2 months

Exp, experimental group, Con control, SD, Sprague–Dawley, BMSCs bone marrow stromal cells, MBMSCs, menstrual blood-derived mesenchymal stem cells, hiMSCs human induced mesenchymal stem cells, hUCMSCs human umbilical cord–derived mesenchymal stem cells, hAMSCs human amniotic mesenchymal, stem cells, hPMSCs human placenta-derived mesenchymal stem cells, hEMSCs human endometrium-derived, mesenchymal stem cells, ADSCs, adipose-derived stem cells, PGS poly(glycerol sebacate), LPS, lipopolysaccharide, PPCNg poly (polyethylene glycol citrate-co-N-isopropylacrylamide) mixed with gelatin, HA-CHO oxidized hyaluronic acid, GEL-ADH hydrazide-grafted gelatin, HA-GEL hyaluronic acid hydrogel.

**Figure 2:**
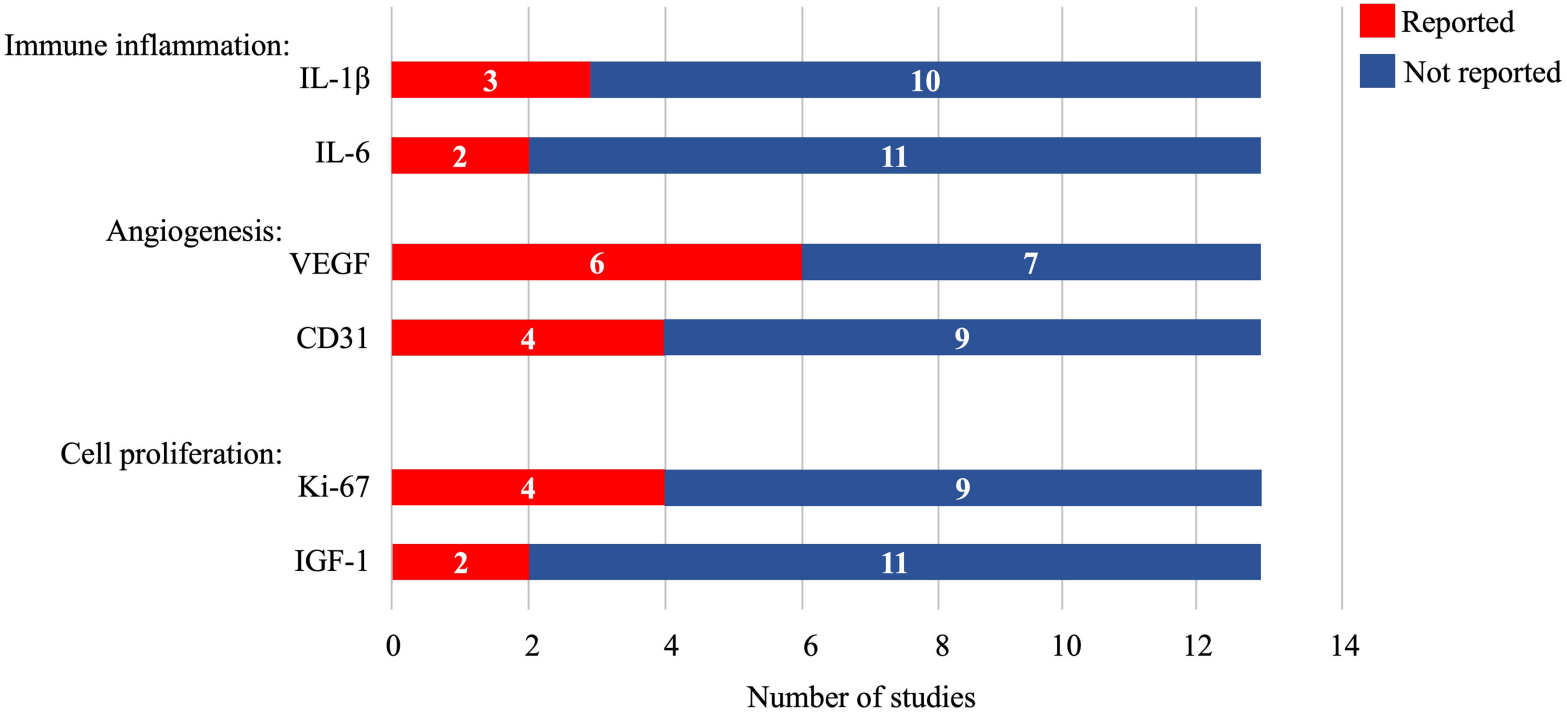
Cytokine and biomarker expression in the included studies. IL-1β, interleukin-1β; IL-6, interleukin-6; VEGF, vascular endothelial growth factor; IGF-1, insulin-like growth factor-1.

The immune system plays an important role in tissue regeneration and determines the speed and outcome of the healing process. It has been suggested that human MSCs have immunomodulatory functions, which can induce a more anti-inflammatory or tolerant phenotype in subpopulations of immune cells by altering their cytokine secretion profile (32). However, the implanted biological scaffolds may intrinsically affect the immune system. Based on these considerations, five studies in this meta-analysis reported the expression of the inflammatory factors IL-1β (three articles) and IL-6 (two articles). Furthermore, angiogenesis, cell proliferation, and differentiation play critical roles in re-epithelializing damaged endometrium. It has been reported that MSCs can promote angiogenesis and maintain cell function by secreting numerous paracrine factors. In this meta-analysis, six studies observed VEGF expression, four observed CD31expression, four observed Ki-67 expression, and two observed IGF-1 expression (**Figure 2**).

### 3.3 Methodological quality of included studies

The CAMARADES tool was used to assess the overall quality of the included studies in 10 aspects. All studies were published in peer-reviewed journals, used appropriate animal models, anesthetized where necessary throughout the study, stated compliance with animal welfare regulations, and declared no potential conflicts of interest. Ten studies (76.92%) described temperature control, while nine (69.23%) randomly allocated animals to treatment or control. However, none of the studies reported sample size calculations, blinded established models, or blinded assessment of the outcomes. The quality scores of 13 studies ranged from 5 to 7 (average 6.46 points). **Table 3** lists the details of the study quality assessment. In summary, 53.85% of the studies were scored as low risk, and 46.15% were at medium risk.

**Table 3:** Quality assessment of included studies based on CAMARADES checklist.

Study	(1)	(2)	(3)	(4)	(5)	(6)	(7)	(8)	(9)	(10)	Score
Yang et al (22)	1	0	1	0	0	1	1	0	1	1	6
Xiao et al (15)	1	1	1	0	0	1	1	0	1	1	7
Chen et al (23)	1	1	0	0	0	1	1	0	1	1	6
Ji et al (18)	1	0	0	0	0	1	1	0	1	1	5
Xu et al (24)	1	1	1	0	0	1	1	0	1	1	7
Hu et al (25)	1	1	1	0	0	1	1	0	1	1	7
Huang et al (26)	1	1	1	0	0	1	1	0	1	1	7
Lin et al (27)	1	1	1	0	0	1	1	0	1	1	7
Lv et al (28)	1	0	1	0	0	1	1	0	1	1	6
Zhang et al (9)	1	1	1	0	0	1	1	0	1	1	7
Liu et al (29)	1	1	1	0	0	1	1	0	1	1	7
Zhao et al (30)	1	1	0	0	0	1	1	0	1	1	6
Wang et al (31)	1	1	0	0	0	1	1	0	1	1	6

(1) Peer-reviewed publication; (2) temperature control; (3) randomly allocated animals to treatment or control; (4) blind established model; (5) blinded assessment of outcome; (6) use of anesthetic on animal model where necessary throughout the study; (7) appropriate animal model; (8) sample size calculation; (9) compliance with animal welfare regulations; (10) statement of potential conflict of interests. A total score of 10, 0–3 are recognized as high risk, 4–6 as medium risk, and 7–10 as low risk.

### 3.4 Risk of bias of included studies

**Figure 3:**
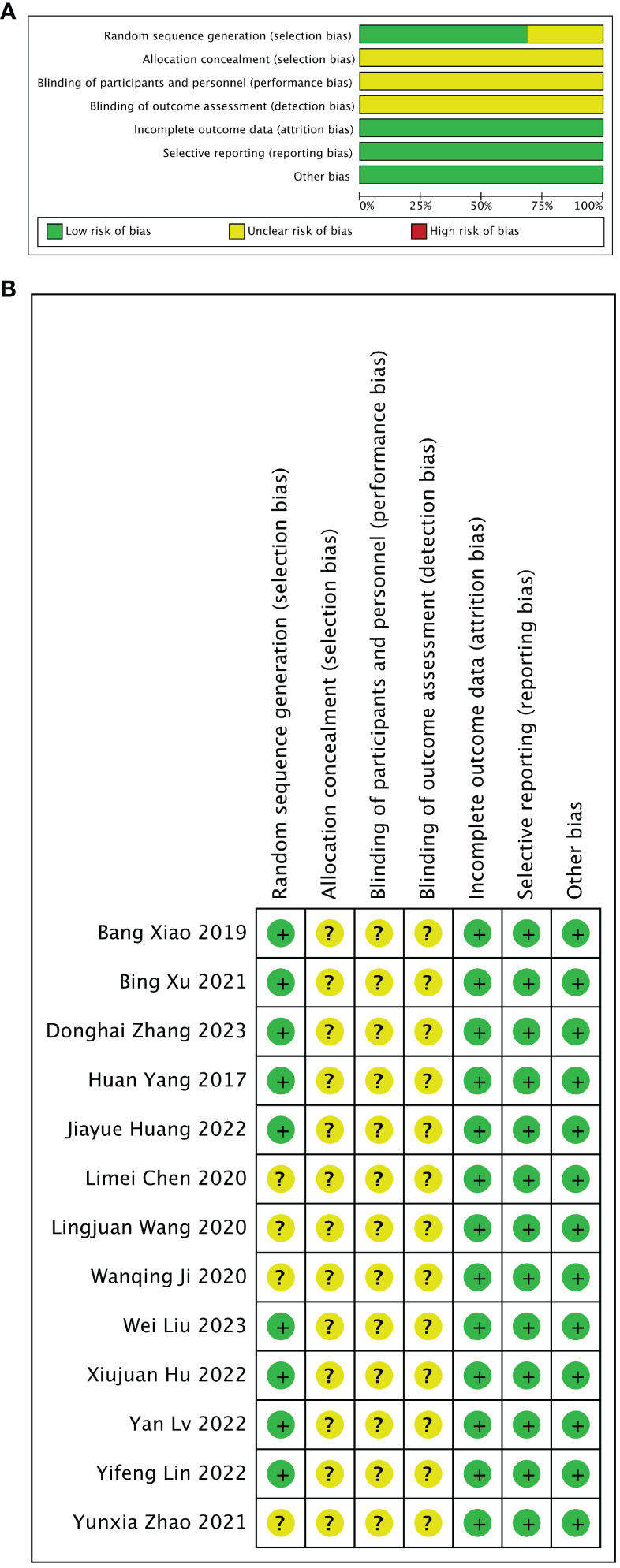
Risk of bias assessed with SYRCLE’s tool. **(A)** Graph showing bias risk. **(B)** Summary of bias risk.

The assessment results of bias risk and methodological suitability of the included studies were presented in **Figure 3**. Nine of the 13 studies divided animals into stem cell-loaded scaffold group and scaffold groups according to random assignment and were therefore judged to have a low risk of selection bias. However, none of the articles mentioned that the studies were conducted by assigning, concealing, blinding investigators, and blinding of the outcome assessment (unclear risk of bias). All studies were free from missing data, selective reporting bias, or other biases (low risk of bias). The methodological quality of the included studies was reliable and acceptable.

### 3.5 Efficacy of stem cell-loaded scaffolds for endometrial injury

#### 3.5.1 Meta 1: Efficacy of stem cell-loaded scaffolds in endometrial thickness

**Figure 4:**
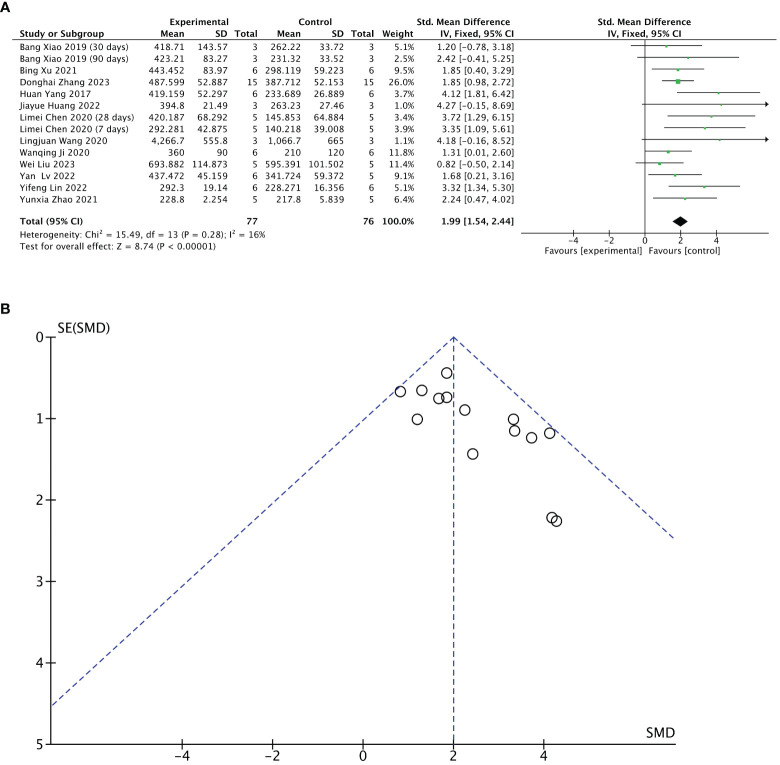
Efficacy of stem cell-loaded scaffold group versus scaffold-only group for endometrial thickness. **(A)** Forest plot displays the mean effect size and 95% confidence interval (CI) for endometrial thickness. **(B)** Funnel plot evaluation of publication bias.

Twelve of 13 included studies reported changes in endometrial thickness after treatment. The stem cell-loaded scaffold and scaffold-only groups had 77 and 76 animals, respectively. I^2^ test (χ^2^ = 15.49, df = 13, P = 0.28; I^2^ = 16% < 50%) indicated low heterogeneity. Therefore, a fixed-effects model was applied in the analysis. Pooled analysis indicated that the stem cell-loaded scaffold groups was superior to scaffold-only groups in increasing endometrial thickness, with a statistically significant difference (SMD = 1.99, 95% CI: 1.54 to 2.44, z = 8.74, P < 0.00001; **Figure 4A**). A funnel plot demonstrated no publication bias existed (**Figure 4B**).

#### 3.5.2 Meta 2: Efficacy of stem cell-loaded scaffolds in the number of endometrial glands

**Figure 5:**
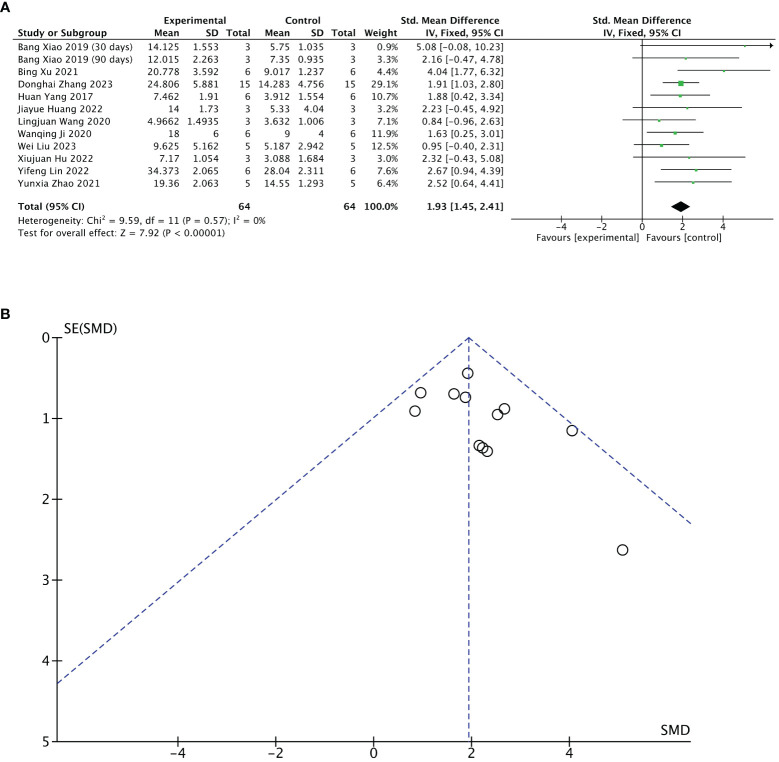
Efficacy of stem cell-loaded scaffold group versus scaffold-only group for the number of endometrial glands. **(A)** Forest plot depicts the mean effect size and 95% confidence interval (CI) for endometrial glands. **(B)** Funnel plot evaluation of publication bias.

Eleven of 13 included studies reported changes in several endometrial glands after treatment. The stem cell-loaded scaffold and scaffold-only groups had 64 animals each. The I^2^ test (χ^2^ = 9.59, df = 11, P = 0.57; I^2^ = 0%) indicated no heterogeneity. Therefore, a fixed effects model was applied. Pooled analysis indicated that the stem cell-loaded scaffold groups was superior to the scaffold-only groups in increasing the number of endometrial glands, with a statistically significant difference (SMD = 1.93, 95% CI: 1.45 to 2.41, z = 7.92, P < 0.00001; **Figure 5A**). A funnel plot revealed no publication bias (**Figure 5B**).

#### 3.5.3 Meta 3: Efficacy of stem cell-loaded scaffolds in fibrotic areas of endometrium

**Figure 6:**
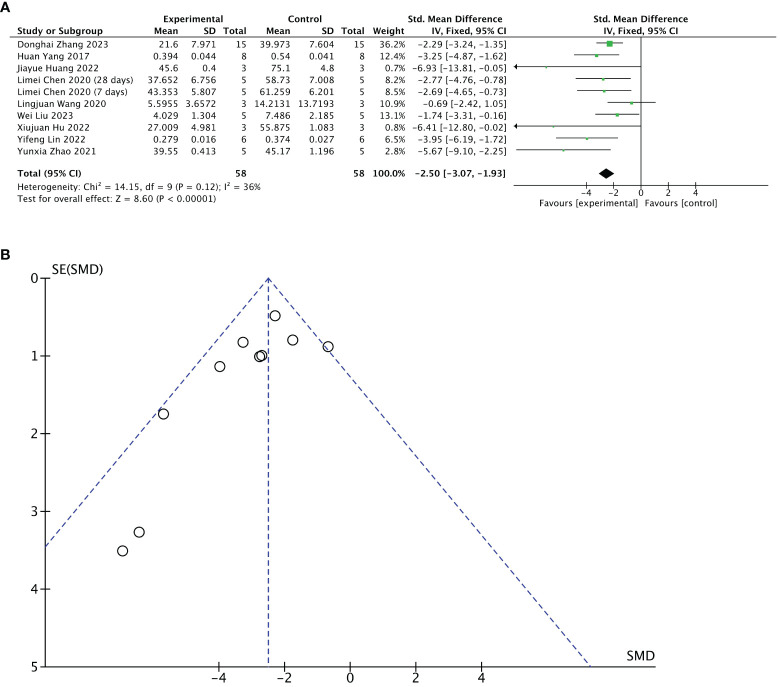
Efficacy of the stem cell-loaded scaffold group versus the scaffold-only group for the fibrotic areas. **(A)** Forest plot illustrates the mean effect size and 95% confidence interval (CI) for the fibrotic area of the endometrium. **(B)** Funnel plot evaluation of publication bias.

**Figure 7:**
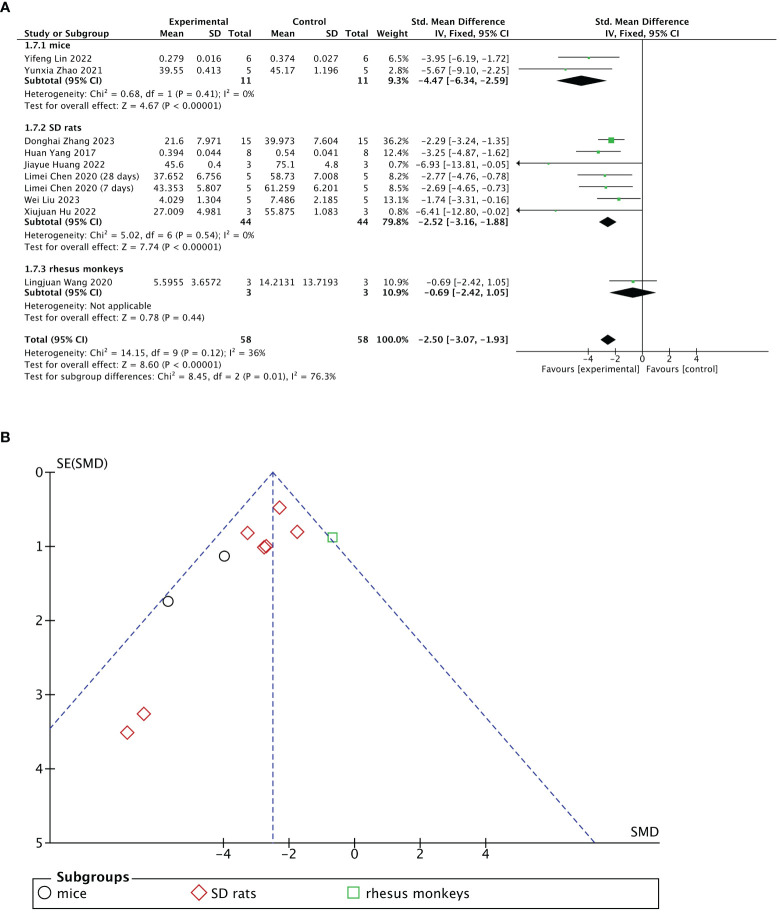
Subgroup analysis of endometrial fibrosis area improvement by animal species [**(A)**, forest plot; **(B)**, funnel plot].

Nine of 13 included studies reported changes in fibrotic areas of the endometrium after treatment. The stem cell-loaded scaffold and scaffold-only groups had 58 animals each. Pooled analysis indicated that the stem cell-loaded scaffold groups had significantly lower fibrotic areas of endometrium than the scaffold-only groups, with a statistically significant difference (SMD = – 2.50, 95% CI: –3.07 to  –1.93, z = 8.60, P < 0.00001; **Figure 6A**). However, the funnel plot revealed that one study in the critical region of the 95% CI (**Figure 6B**). A sensitivity analysis omitting one study at a time indicated potential heterogeneity (χ^2^ = 14.15, df = 9, P = 0.12; I^2^ = 36%) from Wang et al. (31) (**Figure 7B**). Considering that Wang et al. used rhesus monkeys as study subjects, we conducted a subgroup analysis based on animal species. Subgroup analysis demonstrated that stem cells-loaded scaffold groups of mice (SMD = – 4.47, 95% CI: – 6.34 to – 2.59; Z = 4.67, P < 0.00001, **Figure 7A**) and SD rats (SMD = – 2.52, 95% CI: –3.16 to –1.88; Z = 7.74, P < 0.00001, **Figure 7A**) had lower fibrotic areas of endometrium than the scaffold groups, with no heterogeneity within the subgroup (mice subgroup: χ^2^ = 0.68, df = 1, P = 0.41; I^2^ = 0; SD rats subgroup: χ^2^ = 5.02, df = 6, P = 0.54; I^2^ = 0).

#### 3.5.4 Meta 4: Efficacy of stem cell-loaded scaffolds in the number of gestational sacs/implanted embryos

**Figure 8:**
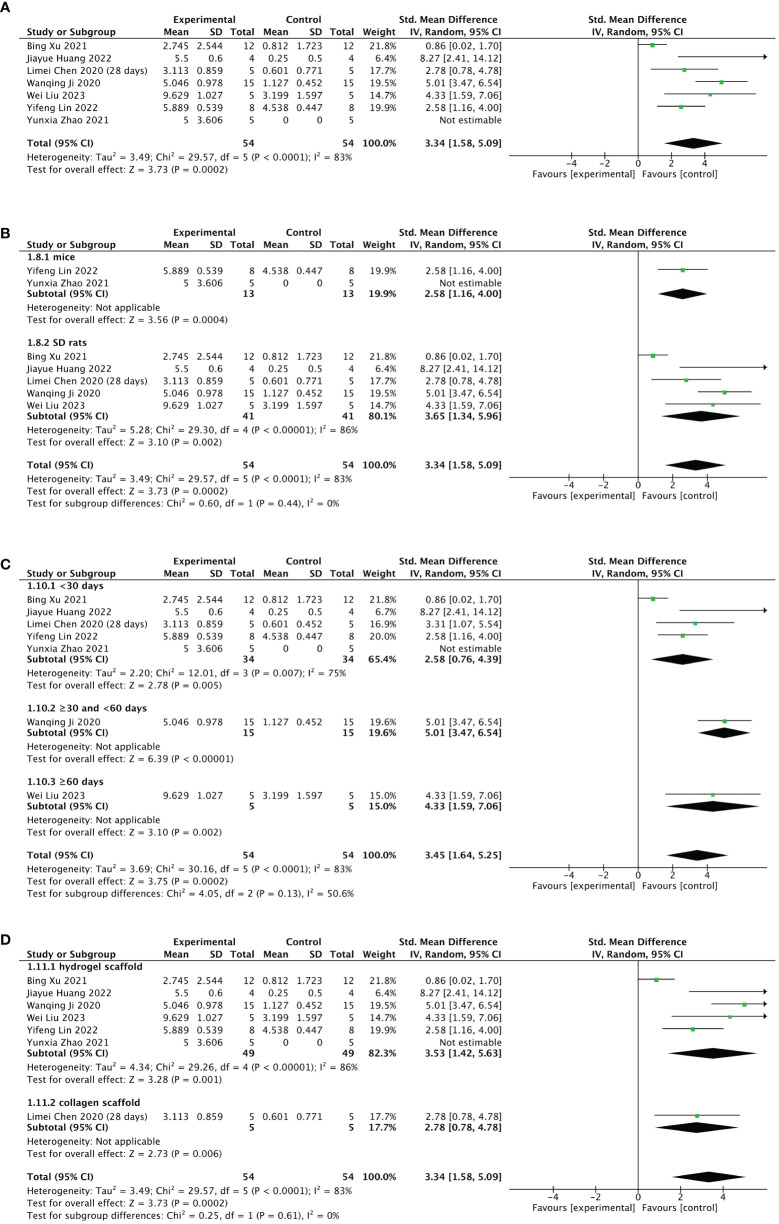
Efficacy of stem cell-loaded scaffold group versus scaffold-only group for the number of gestational sacs/implanted embryos. **(A)** Forest plot indicates the mean effect size and 95% confidence interval (CI) for number of gestational sacs/implanted embryos. **(B–D)**. Subgroup analysis of the number of gestational sacs/implanted embryos according to animal species, treatment time, and scaffold type, respectively.

**Figure 9:**
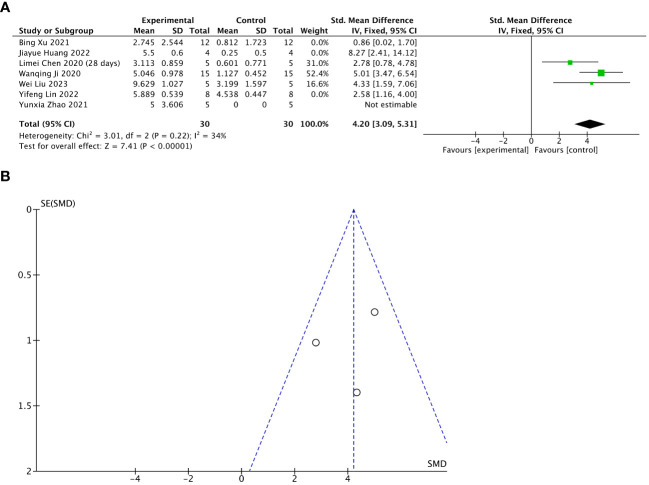
Sensitivity analysis of the number of gestational sacs/implanted embryos. **(A)** Forest plot presents the mean effect size and 95% confidence interval (CI) for number of gestational sacs/implanted embryos. **(B)** Funnel plot evaluation of publication bias.

Seven of 13 included studies reported changes in the number of gestational sacs/implanted embryos after treatment. The stem cell-loaded scaffold and scaffold-only groups each had 54 animals. The I^2^ test (χ^2^ = 29.57, df = 5, P < 0.0001; I^2^ = 83% > 50%) indicated high heterogeneity; therefore, a random effects model was used. The number of gestational sacs/implanted embryos increased markedly in the stem cell-loaded scaffold groups (SMD = 3.34, 95% CI: 1.58 to 5.09, z = 3.73, P = 0.0002; **Figure 8A**). Because the I^2^ value was high, we performed subgroup analysis by animal species, treatment time, and scaffold type. Subgroup analysis by animal species indicated that SD rats (SMD = 3.65, 95% CI: 1.34 to 5.96, z = 3.10, P = 0.002; **Figure 8B**) had a higher effect size than mice (SMD = 2.58, 95% CI: 1.16 to 4.00, z = 3.56, P = 0.0002; **Figure 8B**), without statistically significant difference (P = 0.44, **Figure 8B**). Additionally, neither the treatment time nor the scaffold type had a distinction in the estimation of the effect size (P = 0.13, **Figure 8C**; P = 0.61, **Figure 8D**). These subgroup analyses did not reduce the heterogeneity. Sensitivity analysis revealed that heterogeneity was attributable to the studies of Xu et al. (24), Huang et al. (26), and Lin et al. (27). Excluding these studies reduced heterogeneity to a low level (χ^2^ = 3.01, df = 2, P = 0.22, I^2^ = 34% <  50%; SMD = 4.20, 95% CI: 3.09 to 5.31, z = 7.41, P < 0.00001; **Figure 9A**). A funnel plot indicated no publication bias (**Figure 9B**).

#### 3.5.5 Meta 5: Efficacy of stem cell-loaded scaffolds in immune inflammation

**Figure 10:**
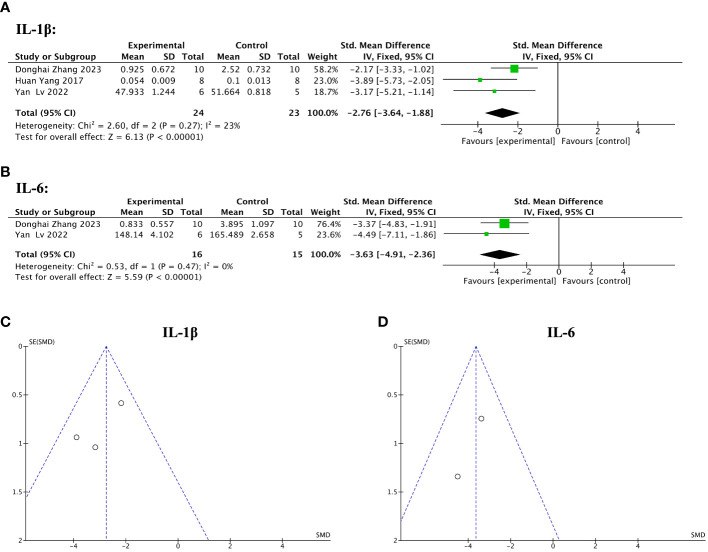
Efficacy of stem cell-loaded scaffolds in immune inflammation. **(A, B)**. Forest plot showing the mean effect size and 95% confidence interval (CI) for interleukin-1β (IL-1β) and interleukin-6 (IL-6), respectively. **(C, D)** Funnel plot evaluation of publication bias for IL-1β and IL-6, respectively.

Of 13 included studies, three reported pre- and post-treatment levels of IL-1β, and two reported pre- and post-treatment levels of IL-6. The pooled analysis indicated that IL-1β (SMD = – 2.76, 95% CI: – 3.64 to – 1.88, z = 6.13, P < 0.00001; **Figure 10A**) and IL-6 (SMD = – 3.63, 95% CI: – 4.91 to – 2.36, z = 5.59, P < 0.00001; **Figure 10B**) were significantly decreased after the treatment with stem cell-loaded scaffolds and heterogeneity was lacking (IL-1β: χ^2^ = 2.60, df = 2, P = 0.27, I^2^ = 23%  <  50%, **Figure 10A**; IL-6: χ^2^ = 0.53, df = 1, P = 0.47, I^2^ = 0, **Figure 10B**). The funnel plot presented no publication bias (**Figures 10C, D**).

#### 3.5.6 Meta 6: Efficacy of stem cell-loaded scaffolds in angiogenesis

**Figure 11:**
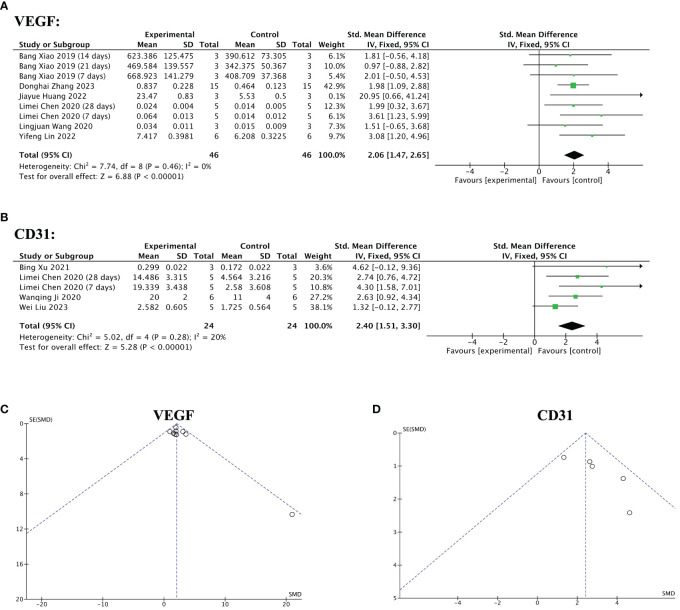
Efficacy of stem cell-loaded scaffolds in angiogenesis. **(A, B)** Forest plot illustrating the mean effect size and 95% confidence interval (CI) for vascular endothelial growth factor (VEGF) and CD31, respectively. **(C, D)**. Funnel plot evaluation of the publication bias for VEGF and CD31, respectively.

Nine of the 13 included studies mentioned angiogenic factors. Among them, five studies reported VEGF levels before and after the treatment. Three studies reported the pre- and post-treatment CD31levels. One study reported changes in VEGF and CD 31 levels. The pooled analysis indicated that stem cell-loaded scaffolds had better angiogenic effects on endometrial injury compared to the treatment with simple scaffolds, as VEGF (SMD = 2.06, 95% CI: 1.47 to 2.65, z = 6.88, P < 0.00001; **Figure 11A**) and CD31 (SMD = 2.40, 95% CI: 1.51 to 3.30, z = 5.28, P < 0.00001; **Figure 11B**) expression was upregulated more in the stem cell-loaded scaffold groups. The funnel plot demonstrated no publication bias (**Figures 11C, D**).

#### 3.5.7 Meta 7: Efficacy of stem cell-loaded scaffolds in cell proliferation

**Figure 12:**
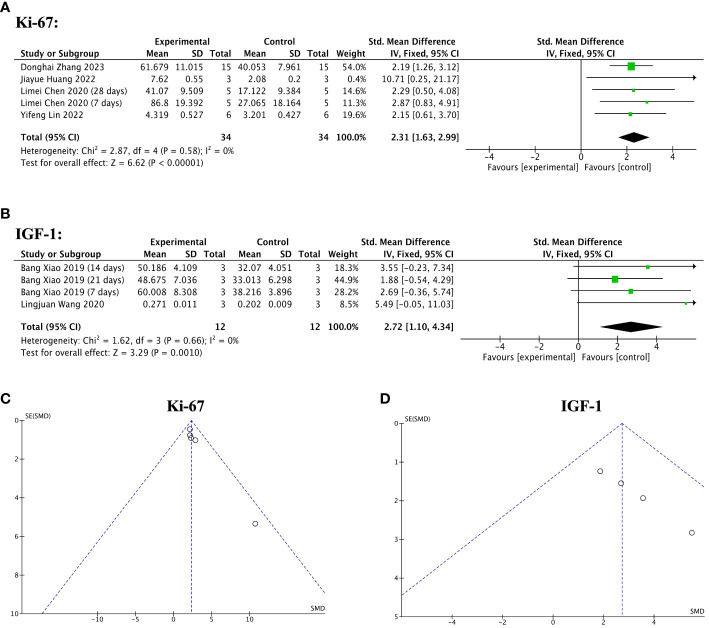
Efficacy of stem cell-loaded scaffolds for cell proliferation. **(A, B)** Forest plot depicting the mean effect size and 95% confidence interval (CI) for Ki-67, and insulin-like growth factor-1(IGF-1), respectively. **(C, D)** Funnel plot evaluation of the publication bias for Ki-67 and IGF-1, respectively.

Four of 13 included studies reported significantly increased Ki-67 levels in the stem cell-loaded scaffold groups compared to the scaffold-only groups (SMD = 2.31, 95% CI: 1.63 to 2.99, z = 6.62, P < 0.00001; **Figure 12A**), with no heterogeneity (χ^2^ = 2.87, df = 4, P = 0.58, I^2^ = 0, **Figure 12A**). Concerning IGF-1 levels, two studies reported that the stem cell-loaded scaffold groups exhibited a higher effect size than the simple scaffold groups (SMD = 2.72, 95% CI: 1.10 to 4.34, z = 3.29, P = 0.0010; **Figure 12B**). The funnel plot depicted no publication bias (**Figures 12C, D**).

## 4 Discussion

### 4.1 Synthesized evidence of stem cell-loaded scaffold therapy in endometrial injury

This meta-analysis synthesized data from 13 studies involving stem cell-loaded scaffolds to treat animal models of endometrial injury. The pooled analysis results were as follows: (1) stem cellloaded scaffold transplantation could promote endometrial regeneration, mainly manifested as thickening of the endometrium and increasing the number of glands; (2) stem cell-loaded scaffold therapy could reduce endometrial fibrosis area, and subgroup analysis revealed a difference in the effect size of animal species in endometrial fibrosis; (3) stem cells-loaded scaffold therapy was generally effective in increasing the number of gestational sacs/implanted embryos, although there was high heterogeneity among studies; (4) stem cell-loaded scaffold therapy exhibited anti-inflammatory, angiogenic, and anti-apoptotic properties in animal models of endometrial injury, contributing to construct the microenvironment. However, the small sample size reduced the robustness of the data.

IUA is usually treated with laparoscopic surgery and adjunctive medication. However, many patients still relapse after treatment. Accordingly, there is an urgent need for more effective and safe treatments to repair the damaged endometrium and improve its function. We explored whether stem cell-loaded scaffold therapy is superior for treating endometrial injury in animals. Endometrial thinning, gland loss, and fibrosis are the main pathological features of IUA (33, 34); thus, we chose endometrial thickness, number of endometrial glands, and fibrosis area of the endometrium as outcome indicators. The ultimate goal of IUA treatment is to restore fertility; therefore, gestational sacs/embryo implantation capacity was adopted to assess endometrial function.

The results of Meta 1, 2, and 3 demonstrate that stem cell-loaded scaffold therapy for endometrial injury effectively increased endometrial thickness and glandular quantity, reduced fibrotic area, and was superior to scaffold-only therapy. One important point to emphasize is that animal species influenced effect size regarding the endometrial fibrosis area. Stem cell-loaded scaffold transplantation had a stronger therapeutic effect in ameliorating endometrial fibrosis in rodents but more so in mouse animal models, whereas this effect was not significant in rhesus monkeys. Different results may be obtained because rhesus monkeys have a menstrual cycle similar to humans, and their physiological functions differ from animal models. Furthermore, the results of Meta 4 indicated a significant increase in the number of gestational sacs/implanted embryos after stem cellloaded scaffold transplantation, with statistically significant heterogeneity. Subsequently, we conducted a subgroup analysis of animal species, treatment time, and scaffold type, but none showed reduced heterogeneity. We found that choosing SD rats as animal models, treating them for one or two months, and using hydrogel scaffolds appeared more effective, although there was no statistical difference. Nevertheless, the number of studies in each subgroup was small, and further investigations are needed to validate these results. We also performed subgroup analyses based on stem cell type, cell species origin, therapeutic dose, and modeling methods; however, the number of studies included in fertility assessment was small, requiring larger, well-designed preclinical studies to explore these issues in-depth.

### 4.2 The underlying mechanism of stem cell-loaded scaffold therapy in endometrial injury

Although the positive role of stem cell-loaded scaffold therapy in endometrial injury has been widely accepted, their therapeutic potential is gradually being explored in endometrial reconstruction. After IUA, changes in the intrauterine environment include inflammation, hypoxia, and dysfunction of neovascularization (35). Inflammation is critical for triggering local tissue fibrosis and loss of normal functions (36). Multiple inflammatory factors, such as IL-1β, IL-6, and TNF-α, increase after endometrial damage (37). Anti-infection and inflammation control are important environmental safeguards to optimize endometrial regeneration. MSC-based therapy has been reported to suppress inflammatory responses during endometrial repair, and its therapeutic mechanism may generate a favorable immune microenvironment via immunomodulatory properties (38, 39). Our analysis compared inflammatory factors (IL-1β and IL-6) expression in the stem cell-loaded scaffold and scaffold-only groups. IL-1β and IL-6 expression levels were significantly lower in the stem cell-loaded scaffold groups than in the scaffold-only groups, suggesting that MSCs combined with scaffold treatment were more effective in inhibiting inflammation.

Revascularization is a key factor in the successful repair of the endometrium. The endometrial vasculature of IUA exhibits high impedance and hypoxia (40, 41). VEGF, the most important proangiogenic factor, can rapidly promote angiogenesis in the early stages of endometrial repair (31), increasing nutrition, oxygen, and hormones at the damage site (42). Micro-vessel density is an objective indicator of angiogenesis and can be measured using the vascular marker CD31 (43). We used VEGF and CD31 to evaluate post-treatment angiogenesis. The data for the stem cell-loaded scaffold groups were higher than the scaffold-only groups, indicating that stem cell-loaded scaffold transplantation exhibited better angiogenesis outcomes.

Cell proliferation and differentiation are critical to promote endometrial re-epithelialization. However, defective endometrial epithelial cells, insufficient endometrial coverage of the uterine cavity, and interstitial cell hyperplasia after endometrial damage result in endometrial fibrosis and proliferation inhibition (44). Ki-67 is a nuclear antigen mainly in proliferating cells, closely related to cell mitosis and proliferation (45). IGF involves various physiological processes, including cell proliferation, differentiation, and metabolism (46). After estrogen activation, IGF regulates the cell cycle and promotes endometrial epithelial cell proliferation (47). Among all studies, four reported ki-67 levels, and two reported IGF-1 levels, suggesting that stem cell-loaded scaffold therapy was more beneficial than the scaffold-only group in increasing cell proliferation.

These results indicate that stem cell-loaded scaffold transplantation repaired endometrial injury and improved the intrauterine microenvironment, possibly through anti-inflammatory, angiogenesis, and maintaining cell proliferation, etc.

### 4.3 Clinical perspective

Only a few clinical trials have used stem cell-loaded scaffolds to treat patients with IUA. A study reported that five patients with severe Asherman syndrome experienced endometrial regeneration, successful pregnancies, and live births after receiving collagen/BMNC scaffold treatment. In this study, collagen/BMNC scaffold was spread on an18F Foley catheter and then placed into the uterine cavity (48). This procedure was conducted after the administration of Progynova for 10 days and continued for 30 days after surgery with the same dose of Progynova. These five patients received ultrasound scans, hysteroscopic inspection, and endometrial biopsies after three post-surgery menstrual cycles. Another phase I clinical trial used a UCMSC-loaded collagen scaffold and hormone replacement therapy to prevent recurrent IUA after separation surgery (10). Three months after treatment, 10 of the 26 patients became pregnant owing to endometrial and vascular regeneration. Among these 10 patients, eight were successfully delivered without obvious birth defects and placental complications. However, none of these trials had a control group; therefore, it is unclear whether separation surgery, collagen scaffold alone, and/or hormone supplementation would yield similar results. Additionally, Zhu et al. (11) conducted a prospective randomized controlled clinical trial with 140 patients with IUA and a two-year follow-up. One of the strengths of this trial is that the participants were randomly assigned to the BMSC-collagen scaffold plus Foley balloon catheter group and only the Foley balloon catheter group. The trial results indicated that the BMSCcollagen scaffold plus Foley balloon catheter group had a higher ongoing pregnancy rate than the simple Foley balloon catheter group. In this study, BMSCs were autologous, and the collagen scaffold was an effective carrier to hold BMSCs in place during the treatment process.

Despite the initial success, significant work must be conducted to translate the fundamental findings of stem cell-scaffold therapy into clinical applications for treating IUA. For instance, considering ethical issues and immune rejection, is it be better to use autologous stem cells or extracellular vesicles? Besides, the dosage and transplantation frequency of stem cell-loaded scaffolds are typically a topic of concern when applied in clinical practice. More clinical studies are needed to determine the optimal dosage and transplantation frequency of stem cell-loaded scaffold therapy to achieve maximum efficacy and minimize patient harm.

### 4.4 Limitations

However, this meta-analysis has several potential limitations when interpreting the results. First, although our study included 13 articles, the sample size was relatively small. Similarly, the number of criteria studies in each subgroup was small in the subgroup analysis. Further studies with larger sample sizes are warranted to provide sufficient evidence regarding the effect of stem cell-loaded scaffold therapy on endometrial injury. Second, our meta-analysis only collected data from animal models; most were SD rats, which cannot accurately simulate the physical conditions of humans with IUA. Randomized controlled trial studies on large animals and humans are needed to increase the results robustness and to guide their application in the clinic. Third, although the included studies were all medium- or low-risk, few articles reported sample size calculation, blind modeling, and blind outcome assessment, which are important methods to avoid bias. We attempted to reduce the bias by independent screening, data extraction, outcome assessment, and risk of bias with at least two blinded reviewers. Additionally, many unresolved areas must be explored: (1) the best therapeutic dose of stem cells and scaffolds, (2) the optimal source of stem cells, (3) the most suitable transplantation method, and (4) rejection reactions and the occurrence of adverse events after transplantation. Thus, more high-quality studies are required to confirm these issues.

## 5 Conclusion

Stem cell-loaded scaffold therapy could rescue the injured endometrium in animal models by increasing the endometrial thickness and gland number, decreasing the fibrous area, controlling the inflammatory response, promoting angiogenesis and cell proliferation, and restoring fertility. Larger animal studies and high-quality randomized controlled human trials are needed for further investigation due to limitations in the quality of evidence.

## Data availability statement

The original contributions presented in the study are included in the article/**Supplementary Material**. Further inquiries can be directed to the corresponding authors.

## Author contributions

Q-YH: Writing – original draft, Conceptualization. H-DZ: Writing – review & editing, Data curation. Q-YS: Supervision, Writing – review & editing. J-HX: Project administration, Writing – review & editing.
