# Supplementary material for: Validity of stem cell-loaded scaffolds to facilitate endometrial regeneration and restore fertility: a systematic review and meta-analysis
Source: Front Endocrinol (Lausanne). 2024 May 23;15:1397783. doi: 10.3389/fendo.2024.1397783 (PMC11153789; doi:10.3389/fendo.2024.1397783)
Supplement: Supplementary file 1 [file Table_1.docx]

Supplementary Material

# Supplementary file1

**PRISMA_2020_checklist**

| **Section and Topic** | **Item #** | **Checklist item** | **Location where item is reported** |
| --- | --- | --- | --- |
| **TITLE** | | |  |
| Title | 1 | Identify the report as a systematic review. | **Line1-3** |
| **ABSTRACT** | | |  |
| Abstract | 2 | See the PRISMA 2020 for Abstracts checklist. | **Line23-57** |
| **INTRODUCTION** | | |  |
| Rationale | 3 | Describe the rationale for the review in the context of existing knowledge. | **Line62-75** |
| Objectives | 4 | Provide an explicit statement of the objective(s) or question(s) the review addresses. | **Line77-115** |
| **METHODS** | | |  |
| Eligibility criteria | 5 | Specify the inclusion and exclusion criteria for the review and how studies were grouped for the syntheses. | **Line134-147** |
| Information sources | 6 | Specify all databases, registers, websites, organisations, reference lists and other sources searched or consulted to identify studies. Specify the date when each source was last searched or consulted. | **Line125-126** |
| Search strategy | 7 | Present the full search strategies for all databases, registers and websites, including any filters and limits used. | **Line128-130，Addition File2** |
| Selection process | 8 | Specify the methods used to decide whether a study met the inclusion criteria of the review, including how many reviewers screened each record and each report retrieved, whether they worked independently, and if applicable, details of automation tools used in the process. | **Line145-147** |
| Data collection process | 9 | Specify the methods used to collect data from reports, including how many reviewers collected data from each report, whether they worked independently, any processes for obtaining or confirming data from study investigators, and if applicable, details of automation tools used in the process. | **Line150-152** |
| Data items | 10a | List and define all outcomes for which data were sought. Specify whether all results that were compatible with each outcome domain in each study were sought (e.g. for all measures, time points, analyses), and if not, the methods used to decide which results to collect. | **Line156-159** |
|  | 10b | List and define all other variables for which data were sought (e.g. participant and intervention characteristics, funding sources). Describe any assumptions made about any missing or unclear information. | **Line153-155** |
| Study risk of bias assessment | 11 | Specify the methods used to assess risk of bias in the included studies, including details of the tool(s) used, how many reviewers assessed each study and whether they worked independently, and if applicable, details of automation tools used in the process. | **Line175-187**  **Line203-204** |
| Effect measures | 12 | Specify for each outcome the effect measure(s) (e.g. risk ratio, mean difference) used in the synthesis or presentation of results. | **Line193-195** |
| Synthesis methods | 13a | Describe the processes used to decide which studies were eligible for each synthesis (e.g. tabulating the study intervention characteristics and comparing against the planned groups for each synthesis (item #5)). | **Line145-147** |
|  | 13b | Describe any methods required to prepare the data for presentation or synthesis, such as handling of missing summary statistics, or data conversions. | **Line161-172** |
|  | 13c | Describe any methods used to tabulate or visually display results of individual studies and syntheses. | **Table 1,Figure1，Line220-221** |
|  | 13d | Describe any methods used to synthesize results and provide a rationale for the choice(s). If meta-analysis was performed, describe the model(s), method(s) to identify the presence and extent of statistical heterogeneity, and software package(s) used. | **Line190-198** |
|  | 13e | Describe any methods used to explore possible causes of heterogeneity among study results (e.g. subgroup analysis, meta-regression). | **Line199-203** |
|  | 13f | Describe any sensitivity analyses conducted to assess robustness of the synthesized results. | **Line202-203** |
| Reporting bias assessment | 14 | Describe any methods used to assess risk of bias due to missing results in a synthesis (arising from reporting biases). | **Line203-204** |
| Certainty assessment | 15 | Describe any methods used to assess certainty (or confidence) in the body of evidence for an outcome. | **Line199-204** |
| **RESULTS** | | |  |
| Study selection | 16a | Describe the results of the search and selection process, from the number of records identified in the search to the number of studies included in the review, ideally using a flow diagram. | **Line220-221,****Figure 1** |
|  | 16b | Cite studies that might appear to meet the inclusion criteria, but which were excluded, and explain why they were excluded. | **Figure 1** |
| Study characteristics | 17 | Cite each included study and present its characteristics. | **Table 1** |
| Risk of bias in studies | 18 | Present assessments of risk of bias for each included study. | **Line268-277,Table 2** |
| Results of individual studies | 19 | For all outcomes, present, for each study: (a) summary statistics for each group (where appropriate) and (b) an effect estimate and its precision (e.g. confidence/credible interval), ideally using structured tables or plots. | **None** |
| Results of syntheses | 20a | For each synthesis, briefly summarise the characteristics and risk of bias among contributing studies. | **Line268-277,Table 2** |
|  | 20b | Present results of all statistical syntheses conducted. If meta-analysis was done, present for each the summary estimate and its precision (e.g. confidence/credible interval) and measures of statistical heterogeneity. If comparing groups, describe the direction of the effect. | **Line 288-294**  **Line 298-304**  **Line 308-311**  **Line 326-332**  **Line 346-352**  **Line 356-363**  **Line 367-372** |
|  | 20c | Present results of all investigations of possible causes of heterogeneity among study results. | **Line290-291**  **Line300**  **Line321-322**  **Line328-329**  **Line351 Line369** |
|  | 20d | Present results of all sensitivity analyses conducted to assess the robustness of the synthesized results. | **Line314-315**  **Line339-341** |
| Reporting biases | 21 | Present assessments of risk of bias due to missing results (arising from reporting biases) for each synthesis assessed. | **Line294-295**  **Line304**  **Line343 Line352-353 Line363-364**  **Line372-373** |
| Certainty of evidence | 22 | Present assessments of certainty (or confidence) in the body of evidence for each outcome assessed. | **Line 288-294**  **Line 298-304**  **Line 308-311**  **Line 326-332**  **Line 346-352**  **Line 356-363**  **Line 367-372** |
| **DISCUSSION** | | |  |
| Discussion | 23a | Provide a general interpretation of the results in the context of other evidence. | **Line377-388** |
|  | 23b | Discuss any limitations of the evidence included in the review. | **Line501-510** |
|  | 23c | Discuss any limitations of the review processes used. | **Line511-513** |
|  | 23d | Discuss implications of the results for practice, policy, and future research. | **Line490-497** |
| **OTHER INFORMATION** | | |  |
| Registration and protocol | 24a | Provide registration information for the review, including register name and registration number, or state that the review was not registered. | **Line 120-121** |
|  | 24b | Indicate where the review protocol can be accessed, or state that a protocol was not prepared. | **None** |
|  | 24c | Describe and explain any amendments to information provided at registration or in the protocol. | **None** |
| Support | 25 | Describe sources of financial or non-financial support for the review, and the role of the funders or sponsors in the review. | **Line545-547** |
| Competing interests | 26 | Declare any competing interests of review authors. | **Line542** |
| Availability of data, code and other materials | 27 | Report which of the following are publicly available and where they can be found: template data collection forms; data extracted from included studies; data used for all analyses; analytic code; any other materials used in the review. | **Line537-539** |

*From:*  Page MJ, McKenzie JE, Bossuyt PM, Boutron I, Hoffmann TC, Mulrow CD, et al. The PRISMA 2020 statement: an updated guideline for reporting systematic reviews. BMJ 2021;372:n71. doi: 10.1136/bmj.n71

For more information, visit:<http://www.prisma-statement.org/>

# Supplementary file 2

**Search strategy**

| **Database** | **Searches** | **Results** |
| --- | --- | --- |
| PubMed | ((("Gynatresia"[Mesh]) OR ((((((((((((((((((((Gynatresias[Title/Abstract]) OR (Asherman Syndrome[Title/Abstract])) OR (Syndrome, Asherman[Title/Abstract])) OR (Asherman's Syndrome[Title/Abstract])) OR (Ashermans Syndrome[Title/Abstract])) OR (Syndrome, Asherman's[Title/Abstract])) OR (Intrauterine Synechiae[Title/Abstract])) OR (intrauterine adhesion[Title/Abstract])) OR (intrauterine synechia[Title/Abstract])) OR (Synechiae, Intrauterine[Title/Abstract])) OR (Uterine Synechiae[Title/Abstract])) OR (uterus synechia[Title/Abstract])) OR (Synechiae, Uterine[Title/Abstract])) OR (uterine atresia[Title/Abstract])) OR (cervical atresia[Title/Abstract])) OR (uterine atrophy[Title/Abstract])) OR (sclerotic endometrium[Title/Abstract])) OR (endometrial sclerosis[Title/Abstract])) OR (endometrial injury[Title/Abstract])) OR (Fritsch syndrome[Title/Abstract]))) AND (("Biocompatible Materials"[Mesh]) OR ((((((((((((((((Biocompatible Material[Title/Abstract]) OR (Material, Biocompatible[Title/Abstract])) OR (Biomaterials[Title/Abstract])) OR (Biomaterial[Title/Abstract])) OR (Bioartificial Materials[Title/Abstract])) OR (Bioartificial Material[Title/Abstract])) OR (Material, Bioartificial[Title/Abstract])) OR (Hemocompatible Materials[Title/Abstract])) OR (Hemocompatible Material[Title/Abstract])) OR (Material, Hemocompatible[Title/Abstract])) OR (Scaffold[Title/Abstract])) OR (Scaffolds[Title/Abstract])) OR (Scaffolding[Title/Abstract])) OR (Scaffoldings[Title/Abstract])) OR (Scaffold Matrix[Title/Abstract])) OR (Scaffold/Matrix[Title/Abstract])))) AND (("Stem Cells"[Mesh]) OR (((((((((((((((Cells, Stem[Title/Abstract]) OR (Cell, Stem[Title/Abstract])) OR (Stem Cell[Title/Abstract])) OR (Progenitor Cells[Title/Abstract])) OR (Cell, Progenitor[Title/Abstract])) OR (Cells, Progenitor[Title/Abstract])) OR (Progenitor Cell[Title/Abstract])) OR (Mother Cells[Title/Abstract])) OR (Cell, Mother[Title/Abstract])) OR (Cells, Mother[Title/Abstract])) OR (Mother Cell[Title/Abstract])) OR (Colony-Forming Unit[Title/Abstract])) OR (Colony Forming Unit[Title/Abstract])) OR (Colony-Forming Units[Title/Abstract])) OR (Colony Forming Units[Title/Abstract]))) | 28 |
| Embase | ('uterus synechia'/exp OR 'asherman syndrome' OR 'asherman`s syndrome' OR 'ashermans syndrome' OR 'cervical adhesions' OR 'cervical synechia' OR 'cervical synechiae' OR 'corporeal adhesions' OR 'endometrial synechia' OR 'endometrium synechia' OR 'intrauterine adhesion' OR 'intrauterine synechia' OR 'intrauterine synechiae' OR 'syndrome, asherman' OR 'synechia endometrii' OR 'synechia, uterus' OR 'traumatic cervical adhesions' OR 'traumatic cervical uterus adhesions' OR 'traumatic corporal adhesions' OR 'traumatic corporal uterus adhesions' OR 'traumatic uterine adhesions' OR 'uterine adhesions' OR 'uterine synechia' OR 'uterus synechiae' OR 'uterus synechia':ab,ti) AND ('scaffold'/exp OR 'scaffolds' OR 'scaffold matrix' OR 'scaffold/matrix' OR 'scaffolding' OR 'scaffoldings':ab,ti OR 'biomaterial'/exp OR 'biocompatible materials' OR 'biologic material' OR 'biological material' OR 'biomaterial':ab,ti) AND ('stem cell'/exp OR 'cell, stem' OR 'precursor cell' OR 'progenitor cell' OR 'stem cells' OR 'stem cell':ab,ti) | 22 |
| Web of Science | (TI=(Asherman Syndrome OR Gynatresia OR Gynatresias OR Syndrome, Asherman OR Asherman's Syndrome OR Ashermans Syndrome OR Syndrome, Asherman's OR Intrauterine Synechiae OR intrauterine adhesion OR intrauterine synechia OR Synechiae, Intrauterine OR Uterine Synechiae OR uterus synechia OR Synechiae, Uterine OR uterine adhesion OR uterine atresia OR cervical atresia OR uterine atrophy OR sclerotic endometrium OR endometrial sclerosis OR endometrial injury OR Fritsch syndrome) OR AB=(Asherman Syndrome OR Gynatresia OR Gynatresias OR Syndrome, Asherman OR Asherman's Syndrome OR Ashermans Syndrome OR Syndrome, Asherman's OR Intrauterine Synechiae OR intrauterine adhesion OR intrauterine synechia OR Synechiae, Intrauterine OR Uterine Synechiae OR uterus synechia OR Synechiae, Uterine OR uterine adhesion OR uterine atresia OR cervical atresia OR uterine atrophy OR sclerotic endometrium OR endometrial sclerosis OR endometrial injury OR Fritsch syndrome)) AND (TI=(Biocompatible Materials OR Biocompatible Material OR Material, Biocompatible OR Biomaterials OR Biomaterial OR Bioartificial Materials OR Bioartificial Material OR Material, Bioartificial OR Hemocompatible Materials OR Hemocompatible Material OR Material, Hemocompatible OR Scaffold OR Scaffolds OR Scaffold Matrix OR Scaffold/Matrix OR Scaffolding OR Scaffoldings) OR AB=(Biocompatible Materials OR Biocompatible Material OR Material, Biocompatible OR Biomaterials OR Biomaterial OR Bioartificial Materials OR Bioartificial Material OR Material, Bioartificial OR Hemocompatible Materials OR Hemocompatible Material OR Material, Hemocompatible OR Scaffold OR Scaffolds OR Scaffold Matrix OR Scaffold/Matrix OR Scaffolding OR Scaffoldings)) AND (TI=(Stem Cells OR Cell, Stem OR Cells, Stem OR Stem Cell OR Progenitor Cells OR Cell, Progenitor OR Cells, Progenitor OR Progenitor Cell OR Mother Cells OR Cell, Mother OR Cells, Mother OR Mother Cell OR Colony-Forming Unit OR Colony Forming Unit OR Colony-Forming Units OR Colony Forming Units) OR AB=(Stem Cells OR Cell, Stem OR Cells, Stem OR Stem Cell OR Progenitor Cells OR Cell, Progenitor OR Cells, Progenitor OR Progenitor Cell OR Mother Cells OR Cell, Mother OR Cells, Mother OR Mother Cell OR Colony-Forming Unit OR Colony Forming Unit OR Colony-Forming Units OR Colony Forming Units)) | 146 |
| Scopus | ( ( TITLE-ABS-KEY ( asherman AND syndrome ) OR TITLE-ABS-KEY ( syndrome, AND asherman ) OR TITLE-ABS-KEY ( asherman's AND syndrome ) OR TITLE-ABS-KEY ( ashermans AND syndrome ) OR TITLE-ABS-KEY ( syndrome, AND asherman's ) OR TITLE-ABS-KEY ( intrauterine AND synechiae ) OR TITLE-ABS-KEY ( intrauterine AND adhesion ) OR TITLE-ABS-KEY ( intrauterine AND synechia ) OR TITLE-ABS-KEY ( synechiae, AND intrauterine ) OR TITLE-ABS-KEY ( uterine AND synechiae ) OR TITLE-ABS-KEY ( uterus AND synechia ) OR TITLE-ABS-KEY ( synechiae, AND uterine ) OR TITLE-ABS-KEY ( uterine AND adhesion ) OR TITLE-ABS-KEY ( uterine AND atresia ) OR TITLE-ABS-KEY ( cervical AND atresia ) OR TITLE-ABS-KEY ( uterine AND atrophy ) OR TITLE-ABS-KEY ( sclerotic AND endometrium ) OR TITLE-ABS-KEY ( endometrial AND sclerosis ) OR TITLE-ABS-KEY ( endometrial AND injury ) OR TITLE-ABS-KEY ( fritsch AND syndrome ) OR TITLE-ABS-KEY ( gynatresias ) OR TITLE-ABS-KEY ( gynatresia ) ) ) AND ( ( TITLE-ABS-KEY ( biocompatible AND materials ) OR TITLE-ABS-KEY ( biocompatible AND material ) OR TITLE-ABS-KEY ( material, AND biocompatible ) OR TITLE-ABS-KEY ( biomaterials ) OR TITLE-ABS-KEY ( biomaterial ) OR TITLE-ABS-KEY ( bioartificial AND materials ) OR TITLE-ABS-KEY ( bioartificial AND material ) OR TITLE-ABS-KEY ( material, AND bioartificial ) OR TITLE-ABS-KEY ( hemocompatible AND materials ) OR TITLE-ABS-KEY ( hemocompatible AND material ) OR TITLE-ABS-KEY ( material, AND hemocompatible ) OR TITLE-ABS-KEY ( scaffold ) OR TITLE-ABS-KEY ( scaffolds ) OR TITLE-ABS-KEY ( scaffold AND matrix ) OR TITLE-ABS-KEY ( scaffolding ) OR TITLE-ABS-KEY ( scaffoldings ) ) ) AND ( ( TITLE-ABS-KEY ( stem AND cells ) OR TITLE-ABS-KEY ( cell, AND stem ) OR TITLE-ABS-KEY ( cells, AND stem ) OR TITLE-ABS-KEY ( stem AND cell ) OR TITLE-ABS-KEY ( progenitor AND cells ) OR TITLE-ABS-KEY ( cell, AND progenitor ) OR TITLE-ABS-KEY ( cells, AND progenitor ) OR TITLE-ABS-KEY ( progenitor AND cell ) OR TITLE-ABS-KEY ( mother AND cells ) OR TITLE-ABS-KEY ( cell, AND mother ) OR TITLE-ABS-KEY ( cells, AND mother ) OR TITLE-ABS-KEY ( mother AND cell ) OR TITLE-ABS-KEY ( colony-forming AND unit ) OR TITLE-ABS-KEY ( colony AND forming AND unit ) OR TITLE-ABS-KEY ( colony-forming AND units ) OR TITLE-ABS-KEY ( colony AND forming AND units ) ) ) | 89 |
